# QuickStats: Expected Number of Births over a Woman’s Lifetime* — National Vital Statistics System, United States, 1940–2018

**DOI:** 10.15585/mmwr.mm6901a5

**Published:** 2020-01-10

**Figure:**
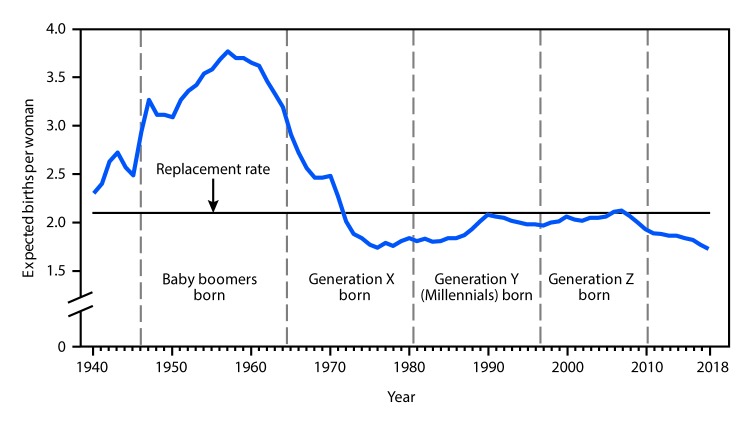
During 1940–2018, the expected number of births a woman would have over her lifetime, the TFR, was highest for women during the post-World War II baby boom (births during 1946–1964). In 1957, the TFR reached a peak of 3.77 births per woman. The TFR generally declined for the birth cohort referred to as Generation X from 2.91 in 1965 to 1.84 in 1980. For the birth cohorts referred to as Millennials (Generation Y) and Generation Z, the TFR first increased to 2.08 in 1990 and then remained generally stable until it began to decline in 2007. By 2018, the expected number of births per women fell to 1.73, a record low for the nation. Except for 2006 and 2007, the TFR has been below the level needed for a generation to replace itself (2.10 births per woman) since 1971.

**Source:** National Vital Statistics System. Birth data, 1940–2018. https://www.cdc.gov/nchs/nvss/births.htm.

